# S-Nitrosoglutathione Reductase Inhibition Regulates Allergen-Induced Lung Inflammation and Airway Hyperreactivity

**DOI:** 10.1371/journal.pone.0070351

**Published:** 2013-07-25

**Authors:** Maria E. Ferrini, Bryan J. Simons, David J. P. Bassett, Matthews O. Bradley, Kevan Roberts, Zeina Jaffar

**Affiliations:** 1 Center for Environmental Health Sciences, Biomedical and Pharmaceutical Sciences, The University of Montana, Missoula, Montana, United States of America; 2 Department of Family Medicine and Public Health Sciences, Wayne State University School of Medicine, Detroit, Michigan, United States of America; 3 SAJE Pharma, Kalispell, Montana, United States of America; Trinity College Dublin, Ireland

## Abstract

Allergic asthma is characterized by Th2 type inflammation, leading to airway hyperresponsivenes, mucus hypersecretion and tissue remodeling. S-Nitrosoglutathione reductase (GSNOR) is an alcohol dehydrogenase involved in the regulation of intracellular levels of S-nitrosothiols. GSNOR activity has been shown to be elevated in human asthmatic lungs, resulting in diminished S-nitrosothiols and thus contributing to increased airway hyperreactivity. Using a mouse model of allergic airway inflammation, we report that intranasal administration of a new selective inhibitor of GSNOR, SPL-334, caused a marked reduction in airway hyperreactivity, allergen-specific T cells and eosinophil accumulation, and mucus production in the lungs in response to allergen inhalation. Moreover, SPL-334 treatment resulted in a significant decrease in the production of the Th2 cytokines IL-5 and IL-13 and the level of the chemokine CCL11 (eotaxin-1) in the airways. Collectively, these observations reveal that GSNOR inhibitors are effective not only in reducing airway hyperresponsiveness but also in limiting lung inflammatory responses mediated by CD4^+^ Th2 cells. These findings suggest that the inhibition of GSNOR may provide a novel therapeutic approach for the treatment of allergic airway inflammation.

## Introduction

Asthma affects 235–300 million patients worldwide and continues to rise in both incidence and morbidity. It is a chronic inflammatory disorder of the lung characterized by airflow obstruction, airway hyperreactivity (AHR) and inflammation in response to exposure to a variety of environmental stimuli including allergens. The pathophysiological features of asthma are associated with the presence in the airways of CD4^+^ T cells and eosinophils, together with goblet cell hyperplasia and mucus hypersecretion, epithelial desquamation and thickening of the submucosa. The role of CD4^+^ Th2 cells and their production of Th2 cytokines, such as IL-4, IL-5, IL-9 and IL-13, have been established in atopic asthma. IL-4 is essential for IgE production, and both IL-9 and IL-13 are important in mucus secretion and AHR, whereas IL-5 promotes eosinophil development, activation and tissue recruitment [1]. CCL11 (also known as eotaxin-1) has also been shown to be a potent and selective eosinophil chemoattractant in humans and is expressed predominantly by epithelial cells [2]. Moreover, CCL11 is important in promoting IL-13-associated allergic lung responses since mice deficient in both IL-5 and CCL11 have an intrinsic defect in IL-13 production by T cells and an impaired development of lung eosinophilia and AHR in experimental asthma [3]. Regulatory T cells, IL-10 and prostanoids have been shown by our laboratory and others to play important roles in regulating Th2-mediated airway inflammation [4], [5], [6], [7], [8].

In addition, nitric oxide signaling pathways have been implicated in the regulation of AHR in asthma [9], [10]. Nitric oxide levels are higher in the exhaled air of patients with asthma than healthy non-asthmatic individuals [11]. The action of nitric oxide is controlled mainly through S-nitrosylation of cysteine residues of proteins to form the more stable S-nitrosothiols [12]. The most abundant S-nitrosothiol in the airway is S-nitrosoglutathione (GSNO), a potent endogenous bronchodilator [13], [14] that may protect against AHR. S-nitrosoglutathione reductase (GSNOR), a member of alcohol dehydrogenase family that is widely expressed in lung tissue [15], has been shown to regulate the level of available endogenous S-nitrosothiols, the bioactive form of nitric oxide, through GSNO catabolism. GSNO is present in high levels in lung lining fluid [13] and has been shown to exert bronchodilatory activity with a 100-fold higher potency than theophylline [16], [17], [18]. The airway levels of GSNO decrease in severe respiratory failure and asthma [14]. Decreased lung GSNO levels are thought to directly contribute to increased AHR during allergic inflammation. Moreover, GSNO degradation has been shown to increase in animal models of allergic asthma [19]. Conversely, mice deficient in GSNOR have increased lung S-nitrosothiols and were protected from the development of AHR. Additionally, GSNO supplementation in an OVA-sensitized and OVA-challenged mice ameliorated AHR [20]. Collectively, these studies suggest that a therapeutic approach in which airway GSNO levels are increased by treatment with GSNOR inhibitors could provide a novel therapeutic approach for reducing allergic inflammation and AHR in asthma and other inflammatory lung diseases.

Due to the accumulating evidence for a role of GSNOR in asthma pathogenesis [10], [21], we used a mouse model of asthma to investigate the effect of a new selective inhibitor of GSNOR, SPL-334, on the inflammatory process. This agent has been shown to exclude GSNO from its binding site and cause an accumulation of S-nitrosothiols inside the cells [22]. We found that SPL-334 administration intranasally during allergic inflammation in mice caused a marked reduction in airway eosinophil and Th2 cell accumulation, mucus secretion and AHR. Thus, GSNOR inhibition may provide a novel approach in limiting allergic airway inflammation and AHR.

## Materials and Methods

### Mice and Ethics Statement

Mice were maintained in microisolator cages and treated in accordance with National Institutes of Health guidelines and the American Association of Laboratory Animal Care regulations and all animal experiments were approved by the University of Montana Institutional Animal Care and Use Committee (IACUC number 052-12). Female or male BALB/c (The Jackson Laboratory, Bar Harbor, ME) and DO11.10 transgenic mice (originally developed by Dr. D.Y. Loh, Howard Hughes Medical Institute, St. Louis, MO) were used throughout the study. DO11.10 transgenic mice were bred under pathogen free conditions in a barrier facility.

### Media

Cells were cultured in RPMI 1640 media supplemented with 10% FBS, L-glutamine (Life Technologies, Carlsbad, CA), penicillin and streptomycin (Life Technologies), HEPES (Life Technologies), sodium pyruvate (Life Technologies), and 2-ME (Sigma Aldrich, St. Louis, MO).

### Preparation of DO11.10 CD4^+^ Th2 Cells

To generate CD4^+^ Th2 effector cells, peripheral lymph nodes obtained from DO11.10 mice were first depleted of CD8^+^ cells using MACS beads (Miltenyi Biotech, Auburn, CA) and incubated (5×10^5^/ml) in complete RPMI media for 4 d in the presence of OVA_323–339_ peptide (1 µg/ml; Mimotopes, San Diego, CA) and IL-4 (2 µg/ml, R&D Systems, Minneapolis, MN) plus mAb anti–IFN-γ (5 µg/ml R4-6A2; American Type Tissue Collection [ATTC], Manassas, VA). After 4 d of incubation, cells were restimulated using culture conditions identical to those previously used, but this time in the presence of exogenous IL-2 (10 ng/ml; R&D Systems) for a further 4 d. On day 8, the cells were depleted of class II^+^ cells by panning by incubating with anti-class II mAb (5 µg/ml M5/114; ATTC) for 30 min, then plate-bound mouse anti-rat IgG (10 µg/ml; Jackson ImmunoResearch, West Grove, PA) for 1 h. Nonadherent (Class II^-^ cells) CD4^+^ Th2 cells (>98% purity) were collected for analysis and transfer experiments. Intracellular staining of CD4^+^ Th2 cells revealed that the majority of the cells were IL-4^+^ and IFN-γ^-^ (<0.1% IFN-γ^+^).

### Transfer of Polarized DO11.10 CD4+ Th2 Cells and OVA Challenge

Eight-day polarized DO11.10 CD4^+^ Th2 cells (6×10^6^ cells/mouse) were adoptively transferred into BALB/c animals by i.v. injection. Mice (four to six per group) were then challenged by exposure in a chamber to aerosolized solutions of OVA (0.5%, Grade V; Sigma-Aldrich) for 20 min/day, over 7 consecutive days using a Wright’s nebulizer. Control mice were exposed to OVA aerosols but did not receive DO11.10 Th2 cells.

### Intranasal Administration of SPL-334

Th2 recipient mice were lightly anesthetized with isofluorane to allow daily intranasal administration of SPL-334 (SAJE Pharma, 0.1 or 1 mg/kg in 30 µl PBS, powder dissolved in PBS by sonication for 5 min, purity of compound confirmed by Mass Spectrometry Core Facility) or sterile PBS alone (vehicle) and the mice were exposed to either OVA aerosols or PBS vehicle (5 hours post treatment with SPL-334 or PBS) for 20 min/day over 7 consecutive days. SPL-334 was initially identified by Sanghani et al as compound C3 (4-{[2-[2-cyanobenzyl)thio]-4-oxothieno{3,2-d]pyrimidin-3(4H)-yl]methyl}benzoic acid) that demonstrated specific inhibition of GSNOR [22], and this report details the chemistry of the compound. Since the biological effects of SPL-334 are likely to be at the level of the airway, an intranasal (local) route was chosen instead of i.p. or oral administration. Treatment by i.p route required higher doses to reduce the allergic inflammatory response and the bioavailability of the compound after oral administration was found to be limited (data not shown).

### Level of Pulmonary Inflammation

Following treatment and OVA inhalation for 7 d (i.e., on day 8), bronchoalveolar lavage was performed (3×0.5 ml PBS) to collect bronchoalveolar lavage fluid (BALF) for analysis. Eosinophil peroxidase (EPO) levels in the bronchoalveolar lavage cells were determined by colorimetric analysis. Cell differential percentages were determined by light microscopic evaluation of Hema3-stained cytospin preparations and expressed as absolute cell numbers. Lung tissue was dispersed by collagenase (Type IV; Sigma-Aldrich), and lung mononuclear cells (LMCs) were isolated by Percoll (Sigma-Aldrich) density gradient for functional analysis.

### Flow Cytometry

FACSAria II (BD Biosciences, San Jose, CA) was used to enumerate the number of CD4^+^KJ1-26^+^ OVA-specific T cells (KJ-126 is an Ab that recognizes I-Ad restricted TCR specific for OVA), CD8^+^ T cells, CD11b^+^Gr-1^+^ neutrophils, F4/80^−^CD11b^+^Siglec-F^+^ eosinophils, or CD11c^+^F4/80^+^ alveolar macrophages in the LMCs or BALF using specific anti-mouse mAbs that include anti-CD4 (GK1.5, allophycocyanin-Cy7–conjugated; BD Biosciences), anti-CD8a (PE-conjugated; BioLegend), anti-mouse DO11.10 TCR (KJ1-26, allophycocyanin-conjugated; eBioscience, San Diego, CA), anti–Gr-1 (Ly-6G/Ly-6C, RB6-8C5, allophycocyanin-Cy7–conjugated; BioLegend), anti-CD11b (M1/70, allophycocyanin-conjugated; BioLegend), anti-CD11c, anti-F4/80 (Alexa Fluor 647; BioLegend) and anti-Siglec-F **(**PE-conjugated, BD Biosciences).

For analysis of intracellular IL-4 or IFN-γ, cells (1×10^6^) were stimulated with 50 ng/ml PMA plus 500 ng/ml ionomycin (Sigma-Aldrich) in the presence of 1 µl of the protein transport inhibitor BD GolgiPlug containing brefeldin A (BD Biosciences) for 4 h at 37°C. Cells (0.5×10^6^) were then blocked using 2.4G2 supernatant (ATCC) and stained with anti-CD4 (GK1.5, allophycocyanin-Cy7–conjugated; BD Biosciences) or isotype control. Following treatment with fixation and permeabilization buffers (BioLegend), cells were intracellularly stained using allophycocyanin-conjugated anti–IL-4 (clone 11B11, from BioLegend) or FITC-conjugated anti–IFN-**γ** (clone XMG1.2, from BioLegend) and analyzed by FACSAria II.

### Measurement of Th2 Cytokine Production

To measure cytokine production, LMCs (1×10^6^ per well) were stimulated with plate-bound anti-CD3 (2 µg/ml 2C11; ATCC) or OVA_323–339_ peptide (1 µg/ml) in 1.0 ml medium in 96-well plates. After 24 h, the supernatants were harvested and IL-4, IL-5, IL-13 and CCL11 production was determined by ELISA (R&D Systems**)**. Cytokines were detected using capture (ATCC) and biotinylated Abs (BD Biosciences), followed by avidin-HRP, then TMB solution (Invitrogen, Carlsbad, CA). Levels of CCL11 in the BALF were also measured by ELISA (R&D systems).

### Lung Histology

Lung tissue was fixed in 4% paraformaldehyde and embedded in paraffin using a Shandon Citadel tissue processor (Thermo Fisher Scientific, Pittsburgh, PA). Microtome sections were cut at 5-µm thickness and stained with H&E using a Shandon Varistain 24–4 (Thermo Fisher Scientific). Alternatively, sections were stained using periodic acid-Schiff (PAS) reagent. PAS stained slides were then imaged with an iCys CompuCyte Laser Scanning Cytometer (LSC, Westwood, Mass) in order to determine the amount of PAS staining and bronchial wall thickness between samples.

### Pulmonary Function Measurement

Respiratory resistance (R_L_, cm H_2_O.s/ml) and dynamic compliance (C_Dyn_, ml/cm H_2_O) were assessed in anesthetized and tracheotomized mice that were mechanically ventilated in response to increasing concentration of methacholine inhalation (1.5–24 mg/ml) using the pulmonary function equipment from Buxco Research Systems.

### Statistical Analysis

Statistical analyses were performed by GraphPad Prism software using nonparametric Mann–Whitney *U* test and data are expressed as means ± SE. A *p* value of 0.05 was considered statistically significant.

## Results

### Airway Inflammation

**Figure 1:**
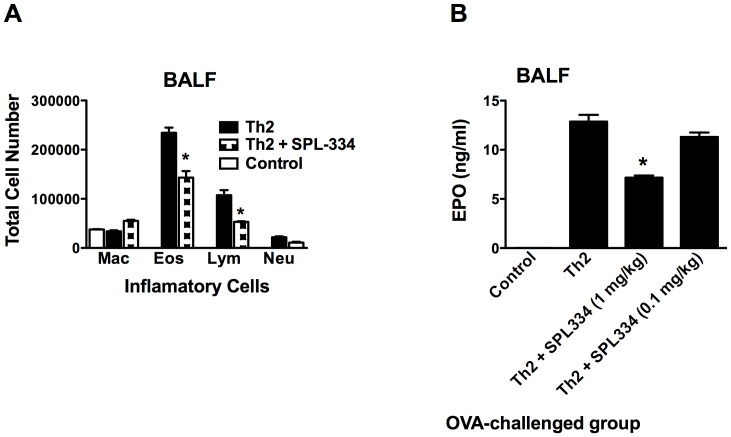
Treatment with the GSNOR inhibitor caused a reduction in allergic airway inflammation. DO11.10 CD4^+^ Th2 cells were adoptively transferred into BALB/c recipient mice that were then exposed to aerosolized OVA for 7 d. Mice were treated intranasally with either the GSNOR inhibitor SPL334 (Th2+ SPL-334, 0.1 or 1 mg/kg daily) or vehicle (Th2). Control mice did not receive Th2 cells but inhaled OVA aerosols. (A) Cell differential counts in the BALF were determined by light microscopic evaluation of stained cytospin preparations. Results are expressed as absolute numbers (per mouse) of lymphocytes (Lym), macrophages (Mac), eosinophils (Eos), and neutrophils (Neu). (B) EPO levels in the BALF from Th2 recipient or control mice were assessed by colorimetric analysis. Results are mean ± SE of four to six individual mice analyzed per group in triplicates and are representative of four independent experiments. *p, 0.05, compared with Th2 group.

**Figure 2:**
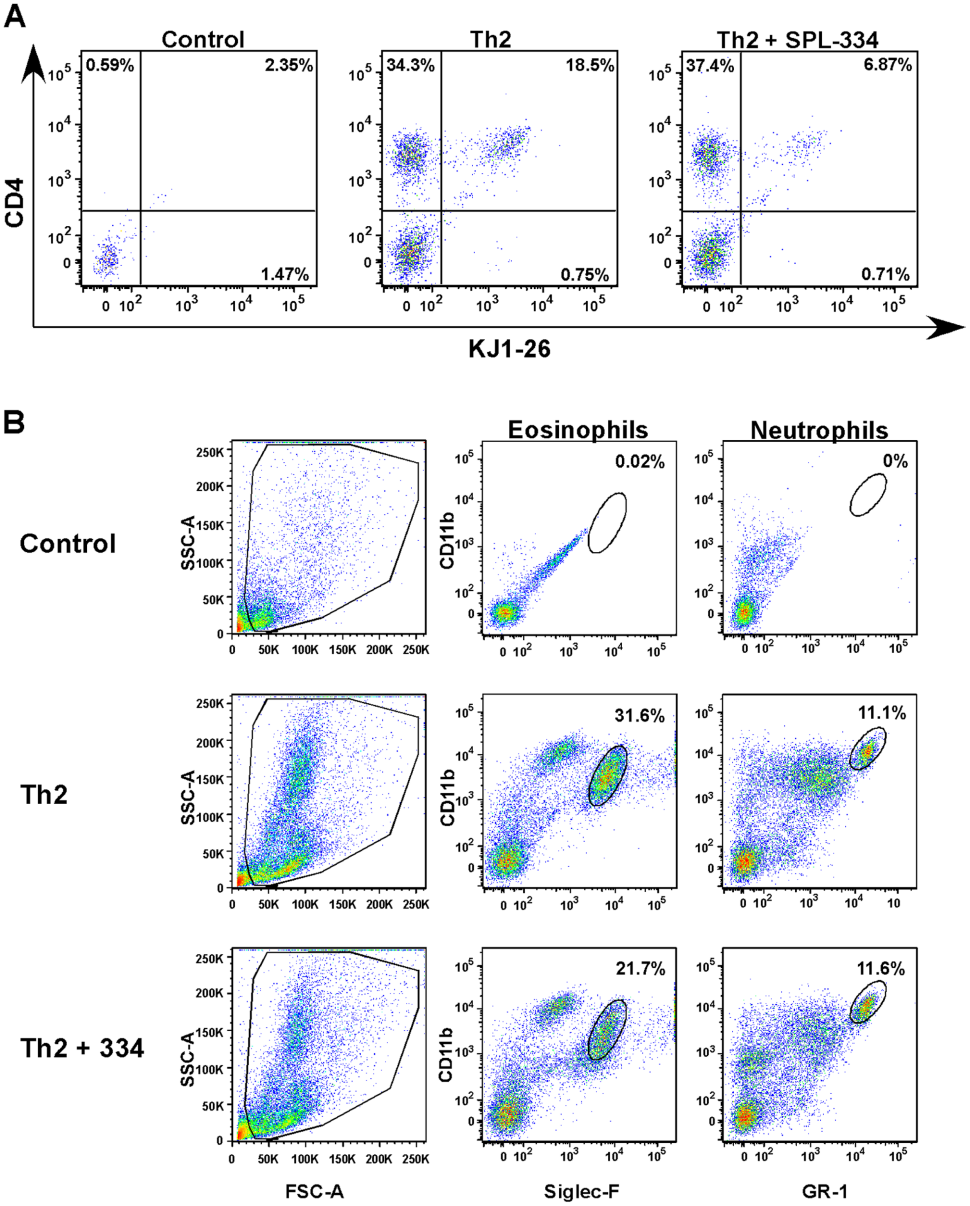
GSNOR inhibitor reduced the number of OVA-specific T cells and eosinophils during allergic airway inflammation. DO11.10 CD4^+^ Th2 cells were transferred into BALB/c mice that were then challenged with aerosolized OVA for 7 d. Mice were treated with either SPL-334 (Th2+ SPL-334) or vehicle (Th2). Control mice did not receive Th2 cells but inhaled OVA aerosols. The number of CD4^+^KJ-126^+^ T cells (A) and CD11b^+^Siglec-F^+^ eosinophils or CD11b^+^GR-1^+^ neutrophils (B) in the BALF following OVA inhalation was determined using flow cytometry. Data are representative of two independent experiments.

To examine the effect of the GSNOR inhibitor, SPL-334, on allergic airway inflammation and whether the inhibitor limited the inflammation mediated by fully differentiated CD4^+^ Th2 cells, we used the passive transfer of Th2 cell model of asthma. In this model, CD4^+^ Th2 cells from DO11.10 TCR transgenic mice are transferred into BALB/c recipient mice that are then exposed to OVA inhalation. Using the adoptive transfer approach, we have previously shown that this model reproduces the cardinal features of allergic asthma (e.g., airway eosinophilic inflammation, mucus production and AHR) but has the advantage that it allows the tracking of OVA-specific T cells, using the anticlonotypic TCR Ab KJ1-26, during the lung inflammatory response [5], [6], [7]. To this end, fully polarized DO11.10 CD4^+^ Th2 cells were generated in vitro, as detailed previously [5], and transferred by i.v. injection into BALB/c mice that were subsequently challenged with aerosolized OVA for 7 d consecutively. The mice were treated daily with either PBS or SPL-334 during the challenge period. Following OVA inhalation, Th2 recipient mice, but not control animals (no Th2 transfer), developed pronounced airway inflammation, characterized by a marked increase in the number of airway lymphocytes and eosinophils (Fig. 1A) and the level of EPO in the BALF (Fig. 1B). Intranasal SPL-334 (1 mg/kg) treatment caused a significant reduction in the influx of lymphocytes and eosinophils into the airways and the level of EPO in the BALF (Fig. 1A and B). A lower dose of SPL-334 (0.1 mg/kg) was ineffective at inhibiting EPO levels in the BALF (Fig. 1B), subsequently 1 mg/kg concentration of the inhibitor was utilized throughout the study. Consistent with the reduction in eosinophilic inflammation, FACS analysis revealed that the proportion and total number of CD4^+^KJ-126^+^ T cells (Fig. 2A) and CD11b^+^Siglec-F^+^ eosinophils (Fig. 2B) in the BALF was markedly reduced following treatment with the GSNOR inhibitor compared to untreated Th2 group. The inhibitor did not affect the levels of CD11b^+^GR-1^+^ neutrophils (Fig. 2B) and CD11c^+^F4/80^+^ alveolar macrophages in the airways (data not shown).

### Peribronchial Inflammation, Mucus Secretion and AHR

**Figure 3:**
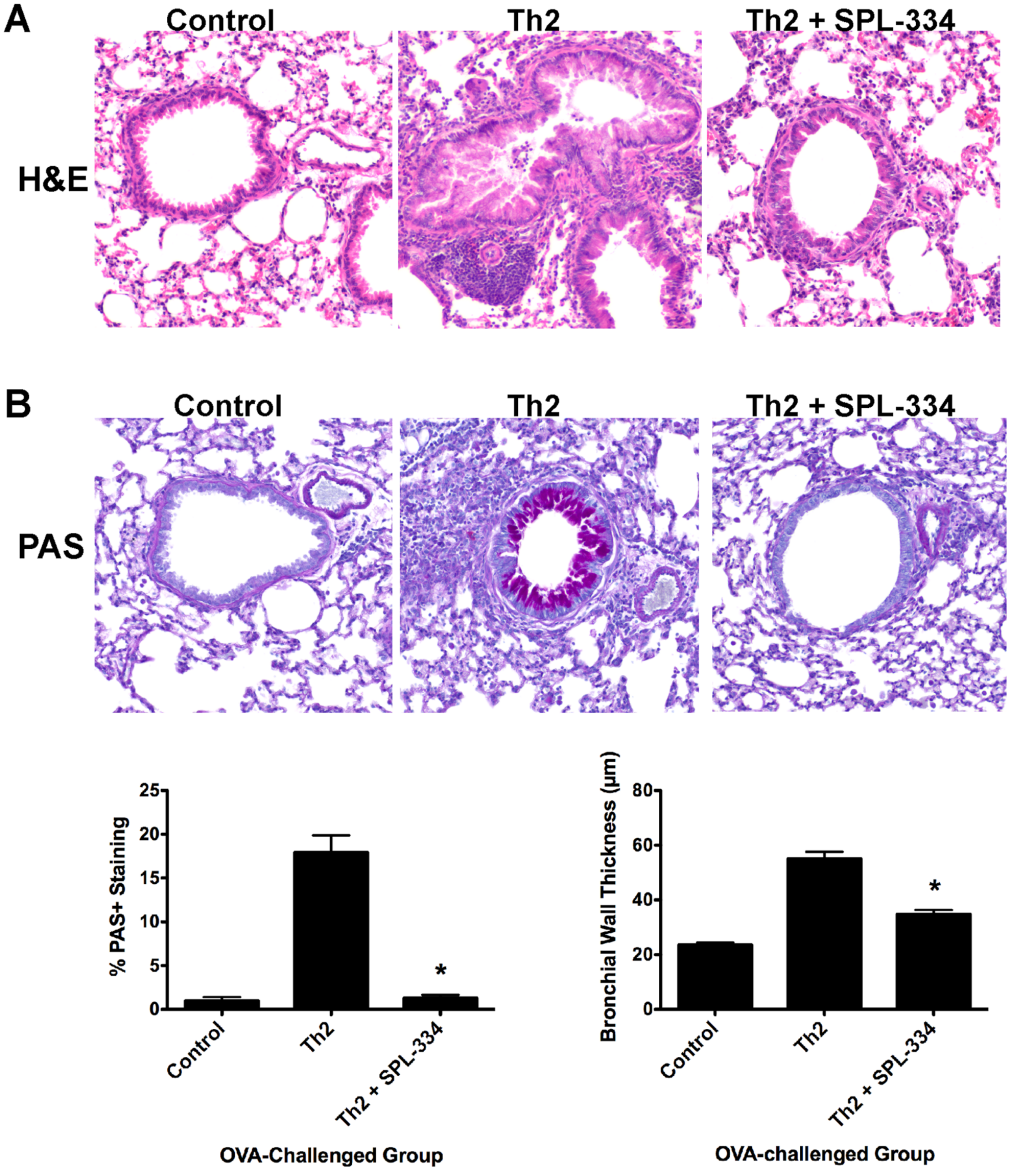
GSNOR inhibitor caused a reduction in peribronchial inflammation and mucus secretion during airway inflammation. DO11.10 CD4^+^ Th2 cells were transferred into BALB/c mice that were then challenged with aerosolized OVA for 7 d. Mice were treated with either SPL-334 (Th2+ SPL-334) or vehicle (Th2). Control mice did not receive Th2 cells but inhaled OVA aerosols. (A) Peribronchial inflammation and (B) mucus production were determined by histological analysis by staining lung tissue sections with H&E or PAS, respectively (20x). PAS staining was expressed as % PAS^+^ area per bronchiole, and bronchial wall thickness expressed in µm. Data are representative of two separate experiments. *p, 0.05, compared with Th2 group.

**Figure 4:**
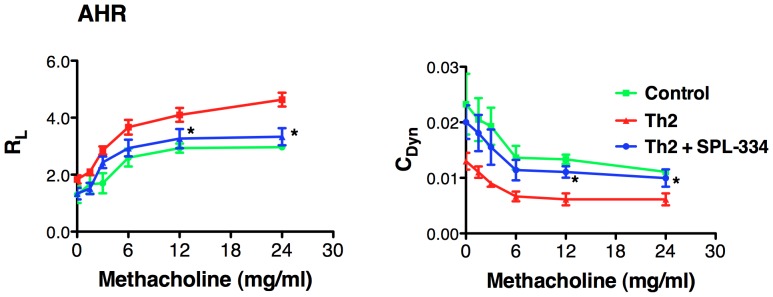
Treatment with the GSNOR inhibitor caused a reduction in AHR . DO11.10 CD4^+^ Th2 cells were transferred into BALB/c mice that were then challenged with aerosolized OVA for 7 d. Mice were treated with either SPL-334 (Th2+ SPL-334) or vehicle (Th2). Control mice did not receive Th2 cells but inhaled OVA aerosols. Lung resistance (R_L_) and dynamic compliance (C_Dyn_) was assessed in anesthetized and tracheotomized mice that were mechanically ventilated in response to increasing concentrations of methacholine inhalation. Data are mean ±SE of ten individual mice analyzed per group. *p, 0.05, compared with Th2 group.

Lung histological analysis (using H&E staining and PAS staining) revealed that Th2 recipient mice had pronounced peribronchial and perivascular inflammation (Fig. 3A) compared to control mice. Moreover, there was a dramatic increase in mucus production and the % PAS staining per bronchiole (Fig. 3B) following OVA inhalation compared to control animals. Both the inflammation and mucus secretion were markedly reduced by treatment with SPL-334 (Fig. 3A and B). The control mice (no Th2 transfer) did not develop any pulmonary inflammation and had no mucus production. The onset of the pulmonary inflammation in Th2 recipients was associated with an increase in airway hyperreactivity (AHR) compared to control mice, as measured by airway resistance (R_L_) and dynamic compliance (C_Dyn_) (Fig. 4). Treatment with SPL-334 reduced the airway resistance and improved the dynamic compliance in mice with lung inflammation (Fig. 4).

### Th2 Cytokine and CCL11 Production

**Figure 5:**
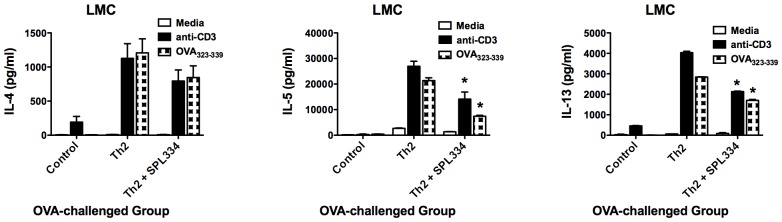
Treatment with the GSNOR inhibitor reduced Th2 cytokine production during allergic airway inflammation. LMCs were obtained from control mice and Th2 recipient mice that have been treated with either SPL-334 or vehicle and inhaled OVA for 7 d. LMCs were stimulated with anti-CD3 (2 µg/ml) or OVA_323–334_ peptide (1 µg/ml) for 24 h and supernatant analyzed for IL-4, IL-5 or IL-13 production by ELISA. Results are mean ±SE of four mice analyzed per group in triplicates and are representative two independent experiments. *p, 0.05, compared with Th2 group.

We next examined the effect of the GSNOR inhibitor on cytokine and chemokine production during allergen-induced airway inflammation. By restimulation of LMCs with OVA_323–339_ peptide, the TCR transgenic model affords a unique approach to examine cytokine production by OVA-specific T cells. LMCs generated from OVA-challenged Th2 recipients produced high levels of IL-4, IL-5 and IL-13 in response to stimulation with OVA_323–339_ peptide or anti-CD3 (Fig. 5). In sharp contrast, lung cells from control mice produced low levels of these cytokines following stimulation (Fig. 5). Importantly, SPL-334 caused a significant reduction in the production of IL-5 and IL-13 by LMCs from OVA-challenged Th2 recipients, though IL-4 production was not significantly decreased.

**Figure 6:**
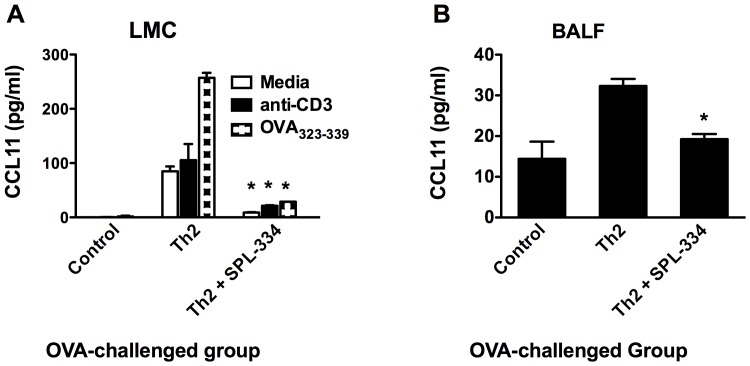
Treatment with the GSNOR inhibitor reduced CCL11 production during allergic lung inflammation. LMCs were obtained from control mice and Th2 recipient mice that have been treated with either SPL-334 or vehicle and inhaled OVA for 7 d. (A) LMCs were stimulated with anti-CD3 (2 µg/ml) or OVA_323–334_ peptide (1 µg/ml) for 24 h and supernatant analyzed for eotaxin production by ELISA. (B) CCL11 levels in the BALF were measured by ELISA. Results are mean ±SE of four to six individual mice analyzed per group in triplicates and are representative two independent experiments. *p, 0.05, compared with Th2 group.

Given that CCL11 plays an important role in eosinophil chemotaxis in asthma, we also examined the effect of GSNOR inhibitor on airway CCL11 production. LMCs from OVA-challenged Th2 recipient mice produced high levels of CCL11 in culture both spontaneously and in response to restimulation with OVA_323–339_ peptide. In mice treated with the GSNOR inhibitor, the level of CCL11 production was markedly reduced (Fig. 6A). LMCs from control animals did not produce eotaxin. Although OVA_323–339_ peptide stimulation (of infiltrating DO11.10 Th2 cells) induced high levels of CCL11 production by the inflamed lung tissue from Th2 recipients, negligible levels were secreted following polyclonal stimulation with anti-CD3. Interestingly, the GSNOR inhibitor significantly reduced both the spontaneous and OVA-induced CCL11 production by the LMCs (Fig. 6A). Consistent with these observations, following OVA inhalation, the level of CCL11 was increased in the BALF of Th2 recipient mice and was significantly reduced by SPL-334 treatment (Fig. 6B).

## Discussion

Work by Que et al using mouse models suggests an important role for GSNOR in the pathogenesis of asthma [10]. The expression of GSNOR in the lung was found to be mainly by airway epithelial cells but macrophages and other infiltrating leukocytes also expressed GSNOR [10]. Following ovalbumin exposure, GSNOR activity increased in the airway lining fluid, possibly as a consequence of its release from damaged epithelial or inflammatory cells [10]. Moreover, elevated GSNOR activity following allergen challenge has been reported to cause increased breakdown of endogenous S-nitrosothiols leading to depleted levels in the human asthmatic airway lining fluid [10], [13]. Notably, decreased concentration of S-nitrosothiols in the airways has been shown to contribute to bronchoconstriction. In support of this, GSNOR knockout mice did not develop bronchoconstriction after administration of methacholine. Therefore, when GSNOR is absent, S-nitrosothiol levels remain elevated, contributing to decreased AHR in the knockout mice [10]. Thus, due to the accumulating evidence for a role of GSNOR in asthma pathogenesis [21], [23], [24], we investigated in the present study the effect of a selective inhibitor of GSNOR, SPL-334, on allergic airway inflammation. Our data demonstrated for the first time that the intranasal administration of SPL-334 significantly reduced Th2-mediated airway inflammation, mucus production and AHR in a mouse model of asthma. Specifically, treatment with SPL-334 caused a marked decrease in the number of OVA-specific T cells and eosinophils present in the BALF of mice that have been adoptively transferred with DO11.10 Th2 cells and exposed to OVA aerosol inhalation. Consistently, there was a marked reduction in the production of Th2 cytokines (IL-5 and IL-13) by LMCs of SPL-334-treated mice following stimulation of OVA-specific T cells with OVA peptide or KJ1-26 antibody, suggesting a decrease in the accumulation of OVA-specific Th2 cells in the lungs during allergic inflammation. In addition, a pronounced decrease in CCL11 production by LMCs and a reduction in CCL11 levels in the BALF were observed in SPL-334-treated mice, compared with vehicle-treated animals. Interestingly, the administration of the GSNOR inhibitor during allergen-induced inflammation also caused a reduction in the spontaneous production of CCL11 by LMCs during culture, suggesting that lung stromal cells may be a major source of CCL11 and that GSNOR inhibition in these cells downregulated their production of CCL11. It is possible that the reduction in CCL11 production in the airways may be a consequence of the lower levels of IL-13 released in the SPL-334-treated mice. Work by Matsukuru et al [25] showed that airway epithelial cells express CCL11 after stimulation with various cytokines (including TNF-α and IL-4) by activating NFκB and STAT6. The C-C chemokine CCL11 is a potent chemoattractant for eosinophils and is thought to play an important role in the pathogenesis of asthma by inducing eosinophil infiltration into the airways. In the present study, given that the administration of the GSNOR inhibitor intranasally caused a marked decrease in CCL11 production in the airways during allergic inflammation, it is likely that this effect was due to the direct action of SPL-334 suppressing NFκB activation in airway epithelial cells and stroma, consequently, downregulating CCL11 production. Such a decrease in CCL11 secretion may have contributed to the reduced eosinophil influx into the airways during the allergic inflammation or, alternatively, may have contributed to the lower levels of IL-13 production and thus IL-13-associated responses [3]. Eosinophil products (granule basic proteins and membrane-derived lipid mediators) are believed to cause much of the bronchial mucosal damage that is thought to ultimately give rise to clinical symptoms of asthma [26].

**Figure 7:**
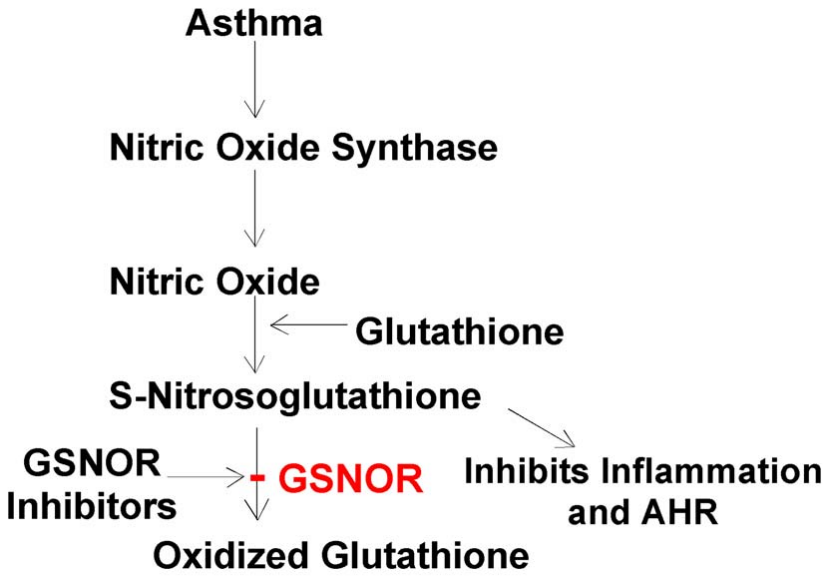
Schematic representation of the anti-inflammatory effects mediated by GSNOR inhibition in asthma. Nitric Oxide produced by a nitric oxide synthase reacts with glutathione to form S-nitrosoglutathione. This reactive molecule promotes bronchodilation and anti-inflammatory effects, and is ultimately degraded to oxidized glutathione and ammonia by S-nitrosoglutathione reductase (GSNOR). We propose that inhibition of GSNOR (which is over-expressed in inflammatory states and asthma) using the GSNOR inhibitor SPL-334 will increase steady-state levels of S-nitrosoglutathione and thus inhibit inflammation and AHR.

The most abundant S-nitrosothiols in the airway is GSNO [13], [14], commonly considered the primary source of bioactive nitric oxide in the body. GSNOR, a member of the alcohol dehydrogenase family, is the major enzyme that catalyzes GSNO metabolism and governs intracellular levels of S-nitosothiols [15]. Recent report showed that GSNOR activity is increased in asthmatic lungs, resulting in diminished S-nitrosothiols and thus contributing to increased AHR [14], [23]. Indeed, GSNO metabolism has been shown to increase in animal models of allergic asthma [19]. Conversely, mice lacking *GSNOR* gene have increased lung S-nitrosothiols and are protected from AHR after methacholine or allergen challenge, suggesting that GSNOR is a crucial modulator of airway tone [10]. GSNO supplementation in an OVA-sensitized and OVA-challenged mice ameliorated AHR [20]. Our findings that GSNOR inhibition reduced AHR further support these studies but also points to a role of GSNOR in allergic inflammatory response in the lungs. SPL-334, identified by Sanghani et al [22] as C3, was shown to exclude GSNO from its binding site and preferentially inhibit GSNOR among the alcohol dehydrogenase isozymes. These authors found that GSNOR activity regulates S-nitrosylation of proteins originating from nitric-oxide synthase activity [22]. Indeed, treatment of RAW 264.7 cells with the GSNOR inhibitor, C3, resulted in the accumulation of nitrosothiols, suggesting that GSNOR actively regulates the turnover and metabolism of S-nitrosothiols inside the cells [22]. Moreover, C3 treatment caused smooth muscle relaxation and suppression of the activation of the transcription factor NF-κB that is consistent with the findings of Matsukuru et al [25]. NF-κB is thought to have a pivotal role in immune and inflammatory responses through the regulation of genes that encode pro-inflammatory cytokines, adhesion molecules, chemokines, growth factors and inducible enzymes, such as cyclooxygenase 2 and inducible nitric oxide synthase (iNOS) [27]. In the present study, given that intranasal treatment with SPL-334 caused a reduction in Th2 cell accumulation, IL-13 and CCL11 production during allergic inflammation, it is possible that the inhibition of GSNOR induced suppression of NF-κB activation in airway epithelial cells and smooth muscle cells as well as in the infiltrating leukocytes, thus downregulating the allergic inflammatory response in the lungs. Importantly, reduction in IL-13 levels using IL-13 deficient mice or IL-13 neutralization strategies have confirmed an essential role for this cytokine in driving major correlates of asthma pathology, including AHR, pulmonary eosinophilia, and mucus secretion [28], [29]. Recently, Sun et al [30] have also identified a novel GSNOR inhibitor and demonstrated that treatment of OVA-challenged mice with the inhibitor (1 mg/kg i.v.) was effective at reducing airway eosinophil infiltration and methacholine-induced bronchoconstriction (using whole body plesthymography). In conclusion, a schematic representation of the proposed anti-inflammatory effects mediated by GSNOR inhibition in asthma is depicted in Fig. 7, where the inhibition of GSNOR (which is over-expressed in inflammatory states and asthma) using GSNOR inhibitors will increase steady-state levels of S-nitrosoglutathione and thus inhibit inflammation and AHR.

In summary, our studies demonstrated that the intranasal administration of SPL-334, a selective inhibitor of GSNOR among the alcohol dehydrogenase isozymes, limits eosinophilic inflammation, mucus production and airway hyperreactivity in a model of allergic asthma. Although further studies are needed, investigation of the effect of GSNOR inhibitors in experimental asthma are warranted, and suggest that increasing airway GSNO levels using selective inhibitors of GSNOR may provide a novel therapeutic target for the treatment of allergic airway inflammation in asthma and other inflammatory lung diseases.
